# Dual functional polyherbal silver nanoparticles for efficient dye degradation and Fe^3+^ ion detection

**DOI:** 10.1039/d5ra08581k

**Published:** 2026-01-19

**Authors:** Ayushma Vyavahare, Priyanshi Dhiraj Shah, Vivek Mishra, Abuzar Shakil Patel, Vasundhara Raina, Sarmita Sanjay Jana

**Affiliations:** a Department of Life Sciences, Parul Institute of Applied Sciences, Faculty of Applied Sciences, Parul University Waghodia Vadodara Gujarat 391760 India sarmita.jana23999@paruluniversity.ac.in vasundhara.raina24479@paruluniversity.ac.in

## Abstract

Silver nanoparticle (AgNP) synthesis using plant extracts has gained significant attention due to its eco-friendly approach and functional versatility. In the present study, stable AgNPs were synthesised using a polyherbal system prepared by employing the extracts of *Rubia cordifolia* (roots), *Terminalia arjuna* (bark) and *Bombax ceiba* (thorns). The combination of chemically diverse phytoconstituents enabled efficient reduction, regulated nucleation and effective surface capping, resulting in well-dispersed, predominantly spherical AgNPs with enhanced colloidal stability. UV-visible spectroscopy confirmed nanoparticle formation through a characteristic SPR peak at 422 nm. HR-TEM analysis revealed predominantly spherical, polycrystalline nanoparticles with an average size of approximately 25 nm. Functionally, the polyherbal AgNPs exhibited excellent catalytic activity, achieving rapid degradation of methylene blue (∼96% within 10 min) and demonstrated sensitive and selective colorimetric detection of Fe^3+^ ions (*R*^2^ = 0.996, over a concentration range of 0.5–3 ppm) with a low detection limit (0.443 ppm). The current findings together with comparative evaluation of previously reported single, dual and multi-plant systems, indicate enhanced functional integration achieved through the present polyherbal strategy.

## Abstract 2

Green synthesis of silver nanoparticles using polyherbal extract for catalytic activity and fe^3+^ detection.

## Graphical abstract

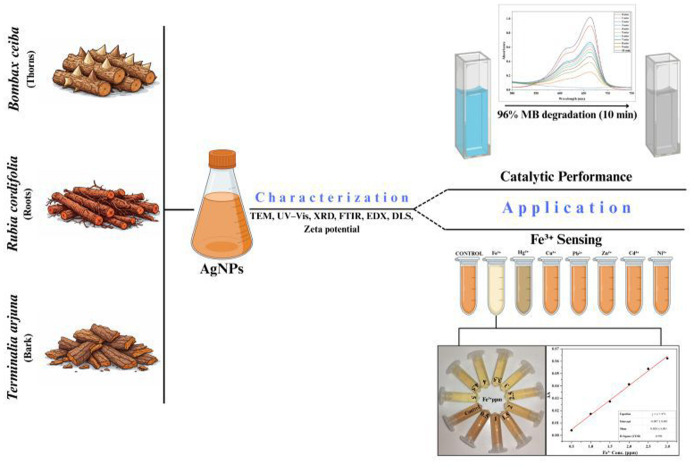

## Introduction

The increasing discharge of synthetic dyes and heavy metal ions into aquatic systems due to industrial and urban activities poses a serious environmental challenge. Dyes such as methylene blue (MB), commonly used in textile, pharmaceutical and printing industries, are persistent, toxic and visually polluting even at low concentrations.^1,2^ Similarly, heavy metal ions such as Fe^3+^, while essential at trace levels, can adversely affect water quality and biological systems when present in excess.^3^ The frequent coexistence of organic dyes and metal ions in wastewater streams necessitates the development of multifunctional materials capable of addressing both classes of contaminants in an efficient and sustainable manner.

Silver nanoparticles (AgNPs) have attracted considerable attention for environmental remediation owing to their high surface area, unique optical properties and excellent catalytic and sensing capabilities. AgNPs act as effective electron-transfer mediators in catalytic reduction reactions and exhibit localized surface plasmon resonance (LSPR), enabling sensitive colorimetric detection of metal ions.^4,5^ However, conventional chemical and physical synthesis routes often involve toxic reducing agents, organic solvents and high energy inputs, limiting their environmental compatibility. Green synthesis of AgNPs using plant extracts has emerged as an eco-friendly alternative that addresses these limitations. Numerous studies have demonstrated that plant-derived phytochemicals such as phenols, flavonoids, tannins, terpenoids, proteins and polysaccharides can act simultaneously as reducing agents, surface capping molecules and stabilizers during NPs formation.^6,7^ These biomolecules regulate nucleation and growth processes, influence particle size and morphology and enhance colloidal stability through electrostatic and steric effects, as evidenced by FTIR, zeta potential and microscopic analyses reported across green AgNPs studies.^8^

Most reported green synthesis studies employ single-plant extracts and several such systems have demonstrated promising catalytic activity toward dye degradation or selective colorimetric sensing of heavy metal ions.^1,9–14^ For example, AgNPs synthesized using *Trigonella foenum-graecum* leaf extract exhibited rapid MB degradation and selective sensing of Fe^3+^ and Hg^2+^ ions.^1^ Similarly, *Terminalia arjuna* bark and leaf extracts have been successfully used to synthesize stable AgNPs with reported photocatalytic degradation of dyes and antimicrobial activity, attributed to their high tannin and flavonoid content.^13,15,16^ These studies establish the feasibility of green AgNPs-based catalysis and sensing using single-plant systems, while also highlighting the influence of extract composition and surface chemistry on NPs performance. In recent years, a limited number of reports have suggested that multi-extract or mixed-plant systems may offer advantages over single-extract approaches by providing a broader spectrum of phytochemicals that collectively influence reduction kinetics, surface passivation and functional performance.^8,17^ Mixed-extract AgNPs have been reported to exhibit improved colloidal stability, enhanced catalytic activity and stronger biological responses compared to NPs derived from individual plant extracts, although detailed mechanistic validation of phytochemical synergy is often beyond the scope of such studies.^10,15,18^

In this context, the present study adopts a literature-guided, assumption-based polyherbal approach rather than a formal hypothesis-testing framework. The selection of a polyherbal extract composed of *Rubia cordifolia*, *Terminalia arjuna* and *Bombax ceiba* was motivated by extensive literature documenting the distinct and complementary phytochemical profiles of these plants and their individual ability to mediate NPs synthesis.^13,16,19–21^. *Rubia cordifolia* extracts, rich in anthraquinones and phenolic compounds, have been previously reported to reduce and stabilize AgNPs with confirmed nanoscale morphology and biological activity.^20^*Terminalia arjuna*, known for its high tannin, flavonoid and triterpenoid content, has been widely used for green AgNPs synthesis, yielding stable NPs with catalytic and antimicrobial properties.^13,16,19^*Bombax ceiba* extracts and flavonoid-rich fractions have also been reported to successfully mediate the synthesis of AgNPs, with FTIR evidence supporting the role of phenolics and terpenoids in NPs reduction and capping^21^ (Table 1).

**Table 1:** Summary of selected plants used for polyherbal extract preparation

Plant name (common name)	Family	Part used	Major phytochemicals	Reported pharmacological properties	Traditional uses	References
*Rubia cordifolia* (Indian Madder/Manjistha)	Rubiaceae	Roots	Anthraquinones (alizarin, purpurin, rubiadin, mollugin), quinones, terpenoids, alkaloids	Antioxidant, anti-inflammatory, cardioprotective, anticancer, antithrombotic, antimicrobial, antifungal, wound-healing	Blood purification, treatment of skin diseases, liver protection and gynaecological disorders	22–30
*Terminalia arjuna* (Arjuna tree)	Combretaceae	Bark	Triterpenoids (arjunin, arjunic acid, arjungenin), tannins (punicalin, castalagin, casuariin), glycosides (arjunetin, arjunolone), flavonoids (baicalein, pelargonidin, kaempferol)	Antioxidant, antimicrobial, cardioprotective, anti-inflammatory, antidiabetic, hepatoprotective, wound-healing	Used as cardiotonic, astringent, demulcent; for ulcers, diabetes, anaemia, liver disorders	31–42
*Bombax ceiba* (Silk Cotton Tree)	Bombacaceae	Thorns	Lupeol, β-sitosterol, mangiferin, tannins, glycosides, naphthoquinone derivatives (gossypol-related)	Antimicrobial, wound-healing, anti-inflammatory, antioxidant, tissue-regenerative	Used for wound healing, skin disorders and as protective agents in inflammatory conditions	43–48

Based on these independent precedents, it was assumed that combining phytochemically diverse plant extracts could favourably influence AgNPs formation, stability and surface functionality. The present study evaluates whether the outcomes of a polyherbal synthesis strategy in terms of AgNPs stability, catalytic efficiency and sensing capability are consistent with expectations derived from prior single-extract and mixed-extract AgNPs literature.^7,8^ This study aimed to develop AgNPs *via* polyherbal route using aqueous extracts of *Rubia cordifolia*, *Terminalia arjuna* and *Bombax ceiba*. The synthesis parameters were optimized to obtain stable, monodisperse NPs, which were characterized to elucidate their structural, surface and colloidal properties. The catalytic performance of the synthesized AgNPs was evaluated using MB degradation as a model reaction and their colorimetric sensing capability toward Fe^3+^ ions was assessed in terms of sensitivity and detection limits.

## Materials and method

### Plant material and chemical reagents

In this study, powdered roots of *Rubia cordifolia* (Manjistha), bark of *Terminalia arjuna* (Arjuna) and thorns of *Bombax ceiba* (Shalmali) were utilized. These plant powders were obtained from Nagarjuna Ayurveda, Nashik, where they are prepared following standardized protocols. The raw materials are sourced from certified suppliers and undergo stringent quality control measures, including chromatographic fingerprinting and contaminant screening. Processing combines traditional Ayurvedic practices with modern extraction techniques to ensure the preservation of active phytoconstituents. Silver nitrate (AgNO_3_) (≥99.8%, AR ACS grade) was purchased from Rankem. Methylene blue (MB) dye was obtained from a local textile supplier. Other chemicals used in this study included cadmium chloride monohydrate (CdCl_2_·H_2_O), copper(ii) sulfate pentahydrate (CuSO_4_·5H_2_O), ferric chloride anhydrous (FeCl_3_), hydrogen chloride (HCl), lead acetate trihydrate (Pb(C_2_H_3_O_2_)_2_·3H_2_O), mercuric chloride (HgCl_2_), nickel sulfate (NiSO_2_), sodium borohydride (NaBH_4_), sodium hydroxide (NaOH) and zinc chloride (ZnCl_2_). All chemicals were used as received without further purification. Furthermore, chemicals and reagents were dissolved in sterile distilled water (DW), unless stated otherwise.

### Preparation of polyherbal extract

An aqueous polyherbal extract was prepared by mixing equal proportions of *Rubia cordifolia* (root), *Bombax ceiba* (thorn) and *Terminalia arjuna* (bark) powders. A total of 1 g of the blended powder was added to 100 mL of DW and heated at 60 °C for one hour with continuous stirring. This process aided in extracting the bioactive constituents and enhancing their dissolution into the aqueous medium. The mixture was then allowed to cool to room temperature, followed by centrifugation at 6000 rpm for 30 min. The resulting supernatant was filtered through Whatman No. 1 filter paper and the clear filtrate was stored in a sterile container at 4 °C until further use. To maximize reduction efficiency, the extract was used within 1 hour of preparation.^13,49,50^

### Synthesis and optimization of polyherbal silver nanoparticles (AgNPs)

A 1% (w/v) aqueous stock solution of AgNO_3_ was prepared in deionized water and used for the green synthesis of AgNPs, with the prepared polyherbal extract serving as both the reducing and stabilizing agent. Optimization for the synthesis was carried out by systematically varying several parameters, including polyherbal extract concentration and its reaction volume, incubation time and pH. The effect of extract concentration and reaction volume was studied in combination, with concentrations adjusted between 1% and 5% and volumes varied from 2 to 10 mL of the selected concentration. Each condition was tested to determine the reaction parameters that produced AgNPs without visible aggregation and maximum stability. For incubation time optimization, reaction mixtures were allowed to proceed for periods ranging from 1 to 24 h, with samples collected at each time point for subsequent UV-Vis analysis. For pH variation, acidic conditions (pH 3–4) were achieved using 0.1 N HCl and basic conditions (pH 8–9) were adjusted using 0.1 N NaOH. Neutral pH was maintained at 7.0 and unaltered pH served as a control. Throughout the optimization, all reactions were performed in a total volume of 50 mL, conducted at room temperature with the reaction flasks covered in aluminium foil and incubated in dark conditions to prevent photo-reduction of Ag^+^. AgNPs formation was initially indicated by a visible colour change, followed by confirmation through UV-Vis spectroscopy in the 400–800 nm range, where the characteristic SPR peak was observed. After the optimal conditions were established, large-scale synthesis of AgNPs was performed and the resulting colloidal suspension was centrifuged at 12 000 rpm for 30 min. The pellet was washed twice with double-distilled water to remove unbound substances and the purified AgNPs were stored in sterile containers at 4 °C for subsequent use.^8,49,51^ Subsequently, the sample was subjected to lyophilization to yield a powdered product.

### Characterization of AgNPs

The optimized AgNPs were characterized using various analytical techniques to assess their formation, stability, morphology and functional groups. Preliminary detection was based on the visible colour change of the reaction mixture, followed by confirmation using UV-visible spectroscopy (Analab Scientific Instrument Pvt. Ltd., ISO 9001:2015 certified) in the wavelength range of 400–800 nm. The surface plasmon resonance (SPR) peak characteristic of AgNPs was recorded both before and after purification, with DW used as the reference blank. For the surface charge and colloidal stability analysis, Dynamic Light Scattering (DLS) and zeta potential measurements (Malvern Instruments Ltd.) were carried out to determine the hydrodynamic size (*H*_D_) distribution and assess the polydispersity index (PDI) of the purified AgNPs. The measurement condition was maintained at a temperature of 25 °C. Furthermore, for the functional group analysis, Fourier Transform Infrared (FTIR) spectroscopy was performed using a Bruker Alpha FTIR spectrometer (Bruker, Germany) to examine the chemical composition and bonding interactions within the AgNPs. The polyherbal powder was finely ground and mixed with potassium bromide (KBr) before measurement, while the AgNPs solution was analysed directly by placing a small drop onto the ATR crystal. Spectral scans were recorded in the range of 400–4000 cm^−1^. Subsequently, Scanning Electron Microscopy (SEM) coupled with Energy Dispersive Spectroscopy (EDS) (Hitachi High-Tech India Pvt. Ltd.) was used to examine the morphology, size and elemental composition of the synthesized AgNPs, confirming the presence of elemental Ag along with plant-derived elements involved in the reduction and capping processes. Here, the AgNPs samples were mounted on conductive Cu tape and dried in a bulb oven at 30–40 °C for 15 min before imaging. X-ray diffraction (XRD) analysis was conducted on the powdered sample using a D6 PHASER instrument (Bruker India Scientific Pvt. Ltd.). Additionally, high-resolution imaging was also performed using a Transmission Electron Microscope (PHILIPS CM200 TEM) operated at an accelerating voltage of 20–200 kV with a resolution of 2.4 Å. For sample preparation, a drop of the colloidal AgNPs suspension was placed onto a 400-mesh copper grid and allowed to dry under infrared (IR) light. TEM analysis, combined with Selected Area Electron Diffraction (SAED), was used to determine the morphology, crystallinity and phase composition of the AgNPs.

### Stability of colloidal AgNPs

The optimized AgNPs solutions were purified, diluted with DW and stored in the dark at 4 °C for 90 days. Their stability was assessed through visual colour observation and UV-visible spectral analysis.

### Catalytic activity – degradation of methylene blue (MB)

A 0.2 M freshly prepared NaBH_4_ solution was prepared in double-distilled water, kept ice-cold and maintained at low temperature throughout the experiment to ensure maximum reactivity. A 10 mM MB stock solution was prepared and appropriately diluted to obtain a 16 µM working solution. The reaction mixture consisted of working MB solution, 200 µL freshly prepared cold NaBH_4_ solution and 30 µL of the pre-determined aliquot of biosynthesized AgNPs colloidal solution. Upon addition of AgNPs, the solution was immediately mixed to ensure uniform dispersion and this point was recorded as *t* = *0* min. Control reactions included MB + NaBH_4_ (without AgNPs) and MB + NaBH_4_ along with poly herbal extract were used to assess reduction. UV-Vis spectra were recorded at 1 min intervals until complete degradation, with primary monitoring at 663 nm (MB *λ*_max_). Spectra were baseline-corrected using DW to remove background interference. The percentage degradation of MB was calculated using eqn (1):1
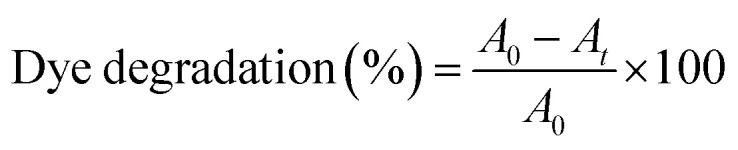
where *A*_0_ is the absorbance at 663 nm at *t* = *0* min and *A*_*t*_ is the absorbance at time *t*. Absorbance was used as a proportional measure of dye concentration under dilute conditions. Kinetic analysis was performed using the pseudo-second-order model^52–54^ (eqn (2)) and the apparent pseudo-second-order rate constant (*k*_2_) obtained from the slope of the 1/*A*_*t*_*versus* time plot and is expressed in Abs^−1^ min^−1^.2
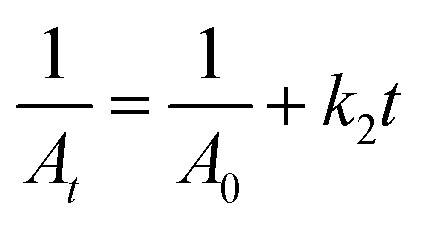


### Colorimetric screening of heavy metal ions

Fresh 10 mM heavy metal stock solutions of CuSO_4_·5H_2_O, CdCl_2_·H_2_O, FeCl_3_, Pb(C_2_H_3_O_2_)_2_·3H_2_O, NiSO_4_, ZnCl_2_ and HgCl_2_ were prepared. In this assay, 100 µL of the biosynthesized AgNPs colloid was taken in a quartz cuvette, followed by the addition of 20 µL respective heavy metal solution. The reaction mixture was made up to a total volume of 2.0 mL with gentle mixing and incubated at room temperature (22–25 °C) for 1 hour in the dark to avoid photoinduced effects. UV-Vis spectra were recorded in the range of 300–700 nm. DW served as the blank and AgNPs dispersed in DW was used as control. Colour changes in the reaction mixture, along with spectral variations, were assessed by monitoring shifts in the SPR peak and changes in absorbance at *λ*_max_; any significant shift or absorbance change was considered a positive detection. The percentage of metal detection was calculated using the formula in eqn (3):3
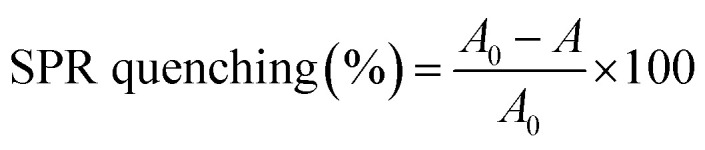
where *A*_0_ is the initial absorbance of the AgNPs solution (control) and *A* is the absorbance after the addition of the heavy metal ion.^55–57^

Following preliminary screening, the most responsive metal ion was selected and quantitative screening was performed over a concentration range of 0.5–5 ppm. A calibration curve was constructed to establish linearity. Additionally, the slope (*S*) of the calibration curve together with the standard deviation (*σ*) of the blank were used to identify the limit of detection (LOD) in ppm^57^ using eqn (4):4
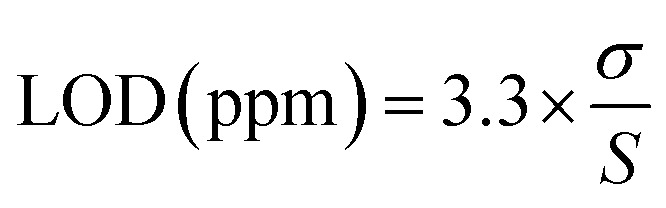


## Result and discussion

### Formation and optimization of polyherbal silver nanoparticles (AgNPs)

#### Effect of extract concentration

**Fig. 1:**
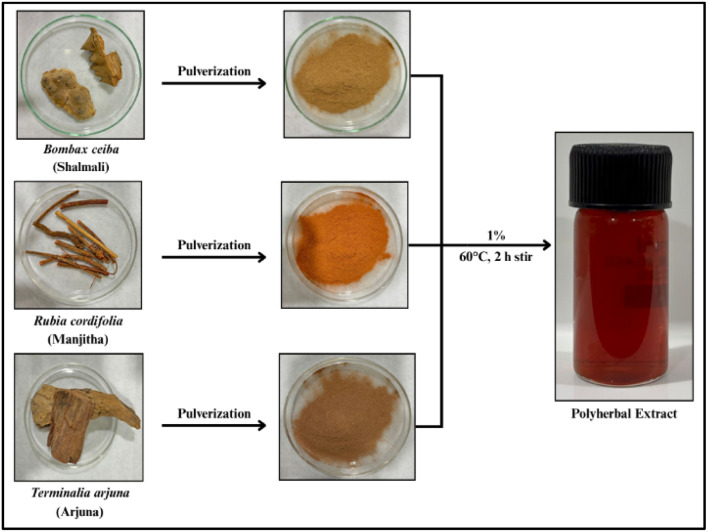
Preparation of polyherbal extract: extract prepared from *Rubia cordifolia* roots, *Bombax ceiba* thorns and *Terminalia arjuna* bark powder.

**Fig. 2:**
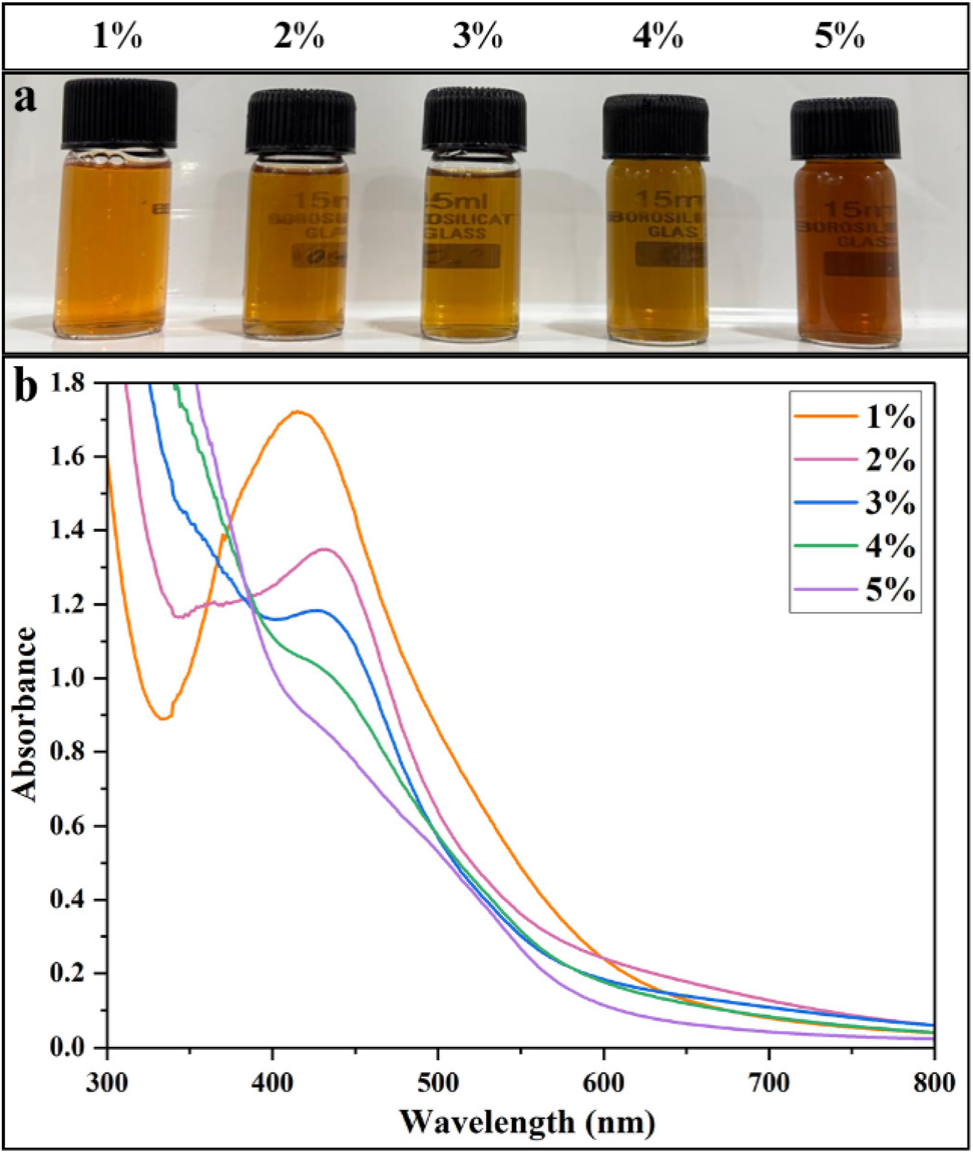
Optimization of AgNPs synthesis with varying plant extract concentrations: (a) visual color changes of the solutions and (b) UV-Vis spectra.

AgNPs were synthesized using 1–5% polyherbal extract (Fig. 1), a clear variation in colour and SPR peak was observed with changes in extract concentration. At 1%, the solution showed a distinct bright-orange colour and a sharp SPR peak at 422 nm with no visible aggregation or precipitation. This indicates that the extract provided polyphenols and flavonoids which primarily act as reducing agents *via* electron donation, while proteins, polysaccharides and terpenoids contribute to nanoparticle stabilization and growth control through capping and steric/electrostatic interactions,^18,58,59^ to efficiently convert Ag^+^ ions into AgNPs.^60^ At 2%, the peak shifted to 430 nm and the orange colour deepened slightly. This colour enhancement, together with the red shift, suggests a higher NPs yield with slightly larger particle sizes, possibly due to faster growth rates or the onset of mild aggregation. At 3% extract concentration, the SPR peak position remained unchanged, although a decrease in absorbance was observed, whereas higher concentrations (4–5%) produced whitish dispersions with no distinct SPR peaks (Fig. 2a and b; SI Table 1). This may be due to the rapid and uncontrolled overcrowding of phytochemicals at the nanoparticle surface at higher extract levels, resulting in large Ag^+^ clusters or precipitates that lack plasmonic behaviour.^61^

#### Effect of plant extract volume

**Fig. 3:**
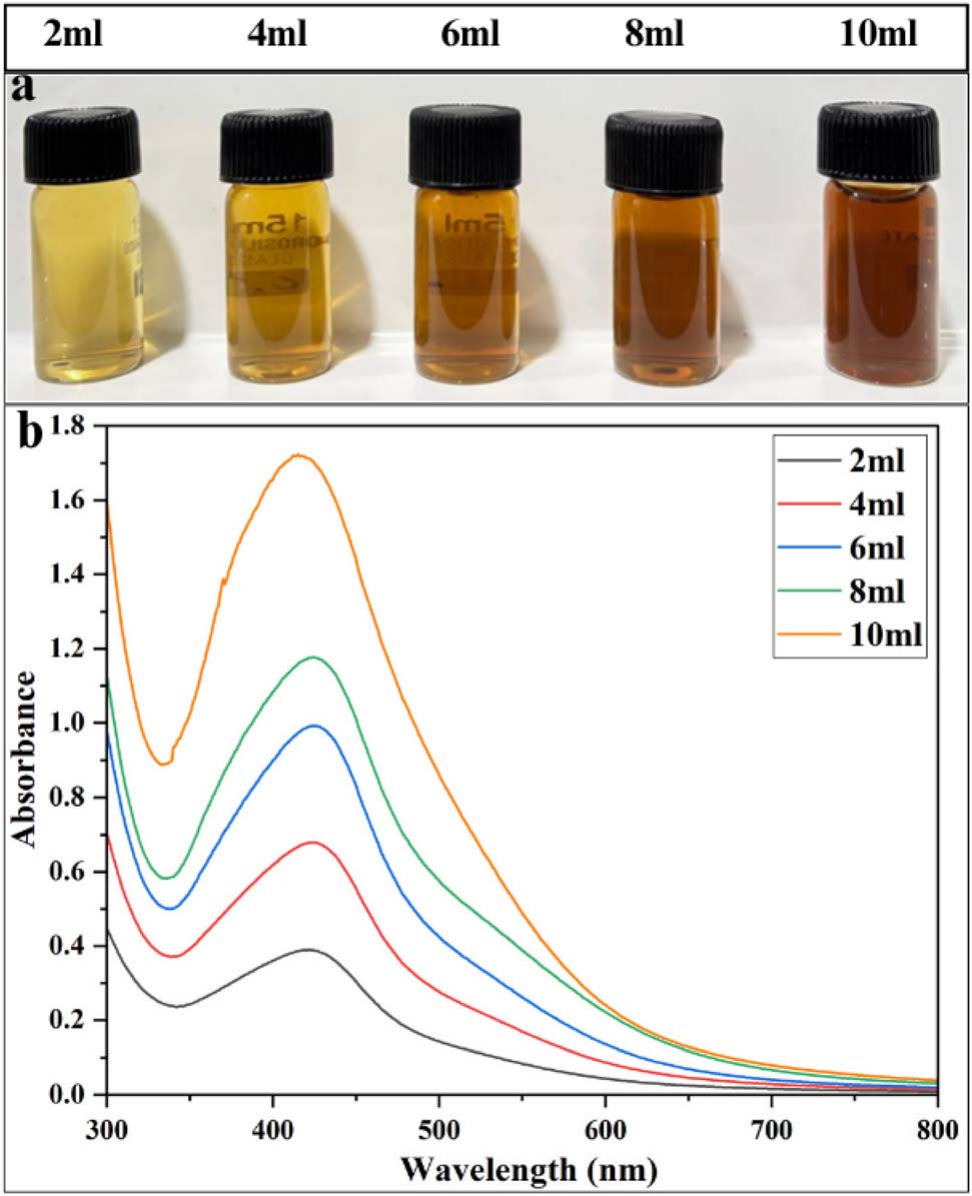
Optimization of AgNPs synthesis with varying volumes of 1% plant extract: (a) visual color changes of the solutions and (b) UV-Vis spectra.

1% plant extract concentration was selected and the effect of varying its volume (2, 4, 6, 8 and 10 mL) on AgNPs synthesis was evaluated. The results showed a clear volume-dependent trend, reflecting the balance between reducing and stabilizing agents in the extract. At lower volumes (2 and 4 mL), synthesis was limited, with minimal colour development, very low absorbance and poorly defined SPR peaks. Increasing the volume to 6 and 8 mL enhanced particle formation, resulting in a noticeable orange colour, sharper peaks (424 nm) and a sequential rise in absorbance. The most pronounced effect was seen at 10 mL, where synthesis was highly efficient, yielding a bright-orange solution and a sharper SPR peak shifted to shorter wavelength (415 nm) (Fig. 3a and b; SI Table 2). This distinct blue-shift suggests the formation of reduced AgNPs with improved electron oscillation properties, due to enhanced capping efficiency at 10 mL volume of 1% extract.^62^ This study clearly shows that optimal amount of bioactive molecules act as effective reducing and stabilizing agents, forming stable surface coatings around the AgNPs.^63^

#### Impact of reaction time

**Fig. 4:**
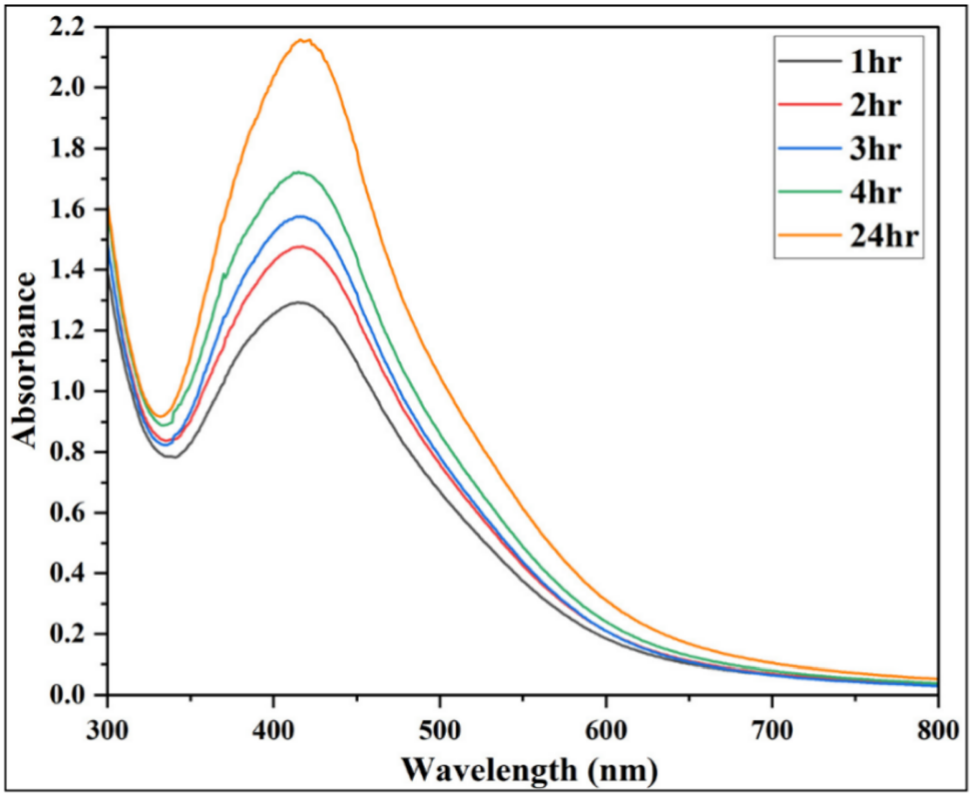
Effect of reaction time on AgNPs synthesis with 10 mL of 1% plant extract: UV-Vis spectra showing absorbance changes over time.

After selecting the optimal plant extract concentration and volume, the next stage of optimization was carried out by tracking the reaction over time. Initially, at 1 hour, a distinct SPR peak appeared at 414 nm with a moderate absorbance, marking the onset of AgNPs formation. As the reaction progressed, the peak gradually red-shifted to 418 nm between 2–8 hours, accompanied by a steady rise in absorbance, indicating increased AgNPs yield. By 24 hours, the SPR peak reached 422 nm (Fig. 4; SI Table 3), with the highest absorbance recorded, signifying complete reduction of Ag^+^ and no further changes were observed thereafter. This red shift reflects the progressive nucleation, increase in particle size and completion of Ag^+^ reduction, which can lead to decrease in energy requirement of excitation of surface plasmon electrons.^62^ The formation of AgNPs within 24 hours is widely reported in the literature, including syntheses using *Acacia cyanophylla*^64^ and *Clinacanthus nutans*.^65^

#### Effect of pH

**Fig. 5:**
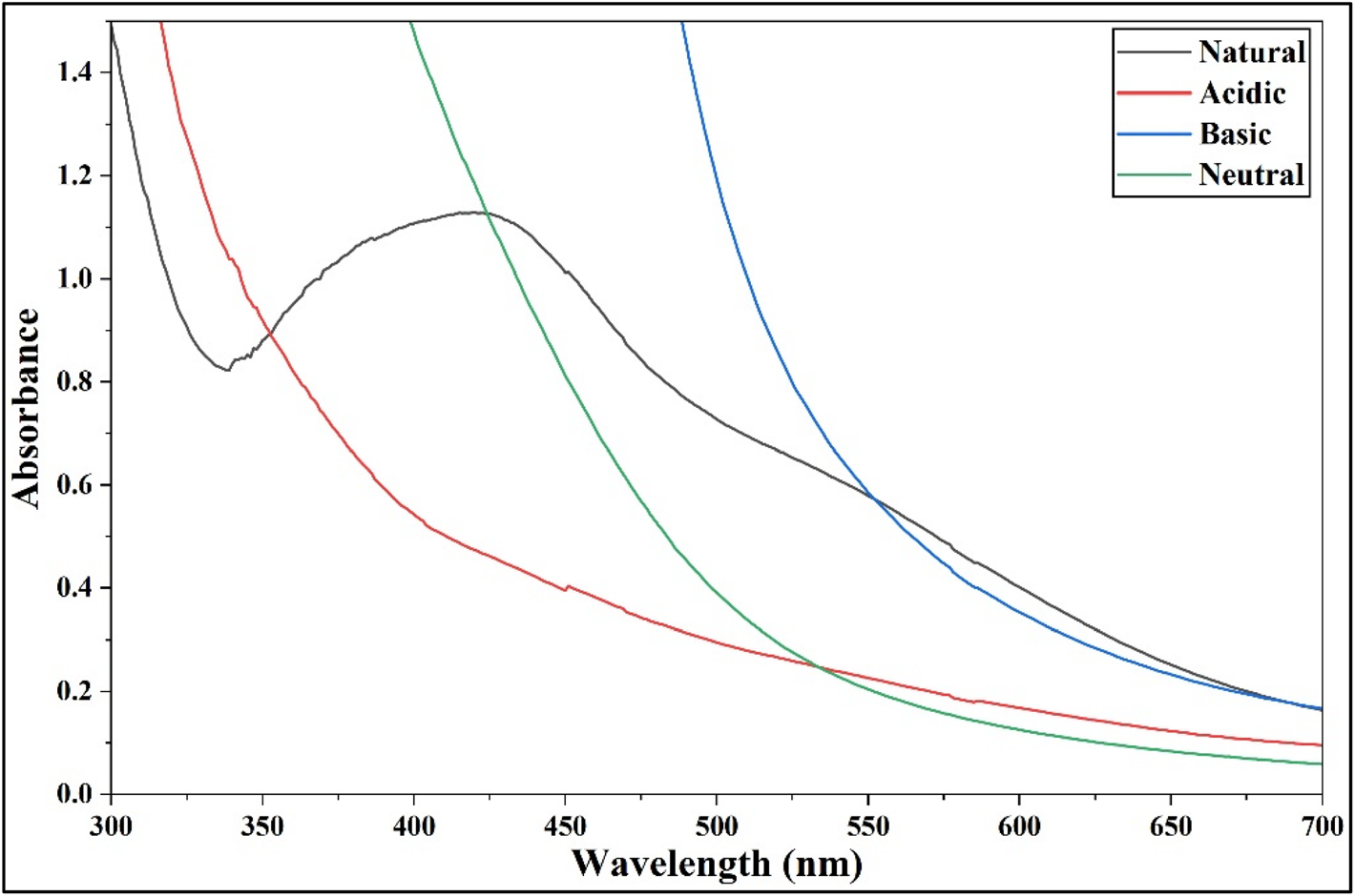
Effect of pH on AgNPs synthesis with 10 mL of 1% plant extract: UV-Vis spectra showing absorbance changes at acidic, neutral, basic and natural pH.

The synthesis of AgNPs was highly sensitive to pH. Extreme acidic (pH 2–4), neutral (pH 7), or basic (pH 9–10) conditions failed to produce AgNPs, likely because they disrupted the plant extract's natural reducing and stabilizing abilities. Maintaining the extract's natural pH (5–6) yielded well-formed, stable AgNPs with a sharp SPR peak at 422 nm (Fig. 5; SI Table 4), as it preserved the optimal ionization of phenolics, flavonoids and proteins, allowing them to efficiently reduce Ag^+^ to Ag^0^ and stabilize them. This highlights that the bioactive compounds work best in their native state, making the natural pH crucial for efficient AgNPs formation.^66^

These findings suggest that the optimum conditions for the synthesis of AgNPs as proposed in the current study include a 1% plant extract concentration, a 10 mL plant extract volume and an incubation period of 24 hours with natural pH 5–6. For purification, centrifugation at 12 000 rpm for 30 min was performed, followed by two washes with distilled water to ensure purity and removal of unbounded components.

### Characterization

#### Ultraviolet-visible spectrophotometry

**Fig. 6:**
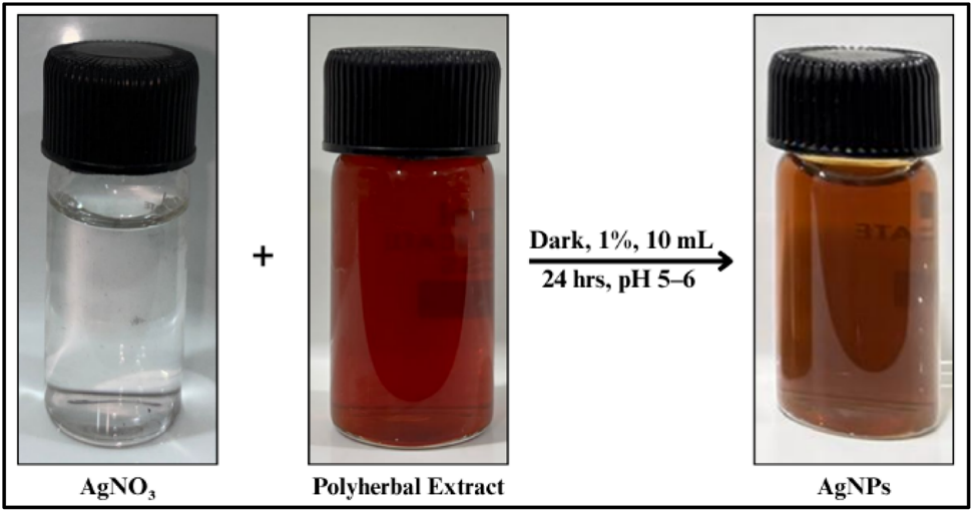
Synthesis of AgNPs under optimized conditions: AgNPs formed using optimized extract concentration, volume, pH and reaction time, showing stable nanoparticle formation.

**Fig. 7:**
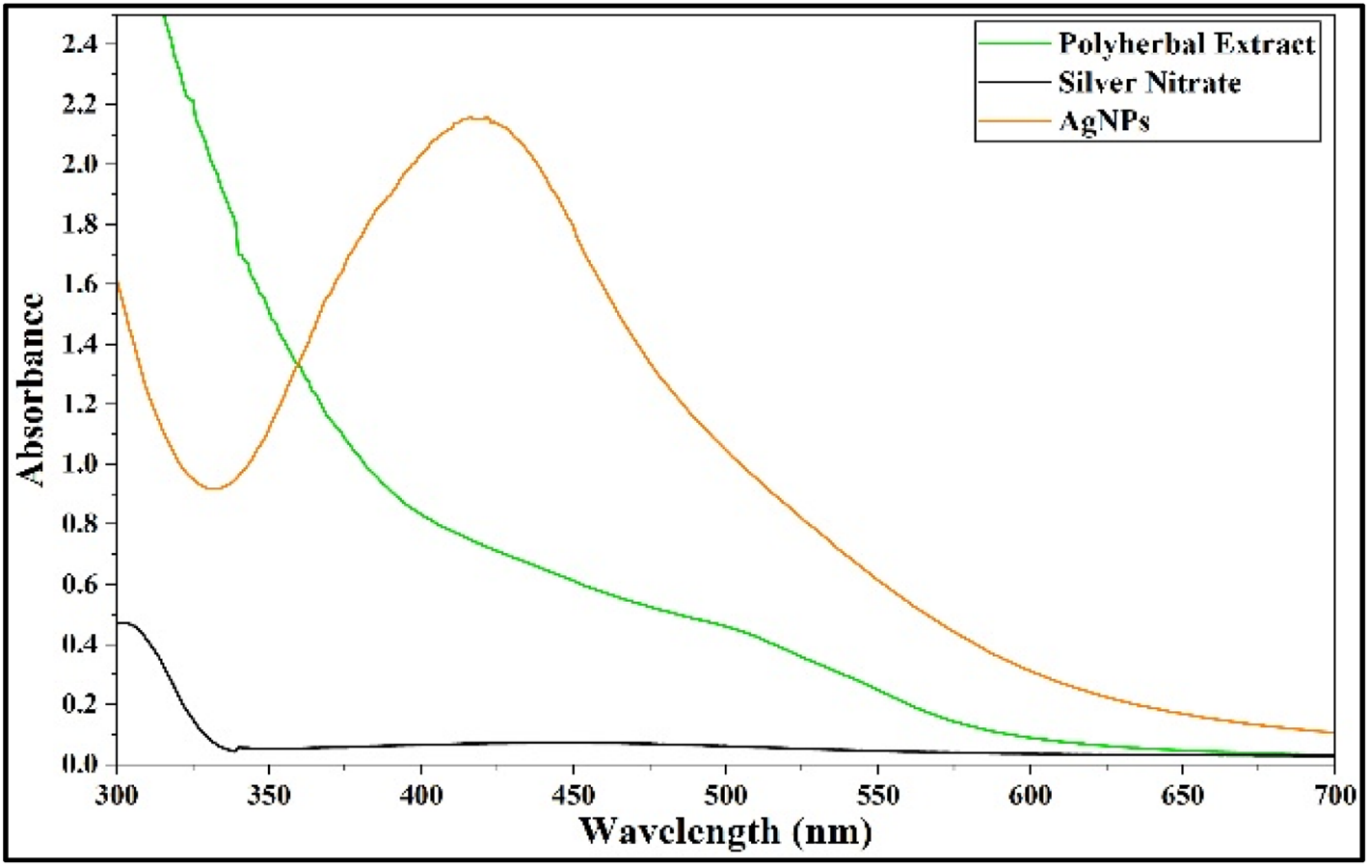
UV-Vis absorbance spectrum of AgNPs: characteristic peak at 422 nm confirms nanoparticle formation.

Following the standardized optimum conditions *i.e.*,10 mL volume of 1% polyherbal plant extract and an incubation period of 24 hours at natural pH 5–6, AgNPs were synthesised and a sharp absorption peak at 422 nm with a distinct bright-orange colour confirmed the same (Fig. 6 and 7; SI Table 5). According to Mie's theory, spherical metal NPs exhibit isotropic behaviour, showing uniform optical properties in all directions, which is displayed as a single, distinct SPR band in their absorption spectrum, whereas anisotropic shapes can produce multiple bands depending on their geometry.^67,68^ In accordance with, previous reports have also confirmed that AgNPs with SPR peaks between 410–450 nm are generally spherical, with particle sizes ranging from 2 to 100 nm (SI Table 11). Based on this, the single SPR peak observed in the present study suggests that the synthesized AgNPs are spherical with uniform particle size distribution. The absorption band observed at 422 nm closely matches the value reported by previous studies of synthesized green AgNPs from *Ocimum basilicum*,^69^*Calotropis gigantea*^70^ and *Pimenta dioica*.^71^

#### Zeta potential

**Fig. 8:**
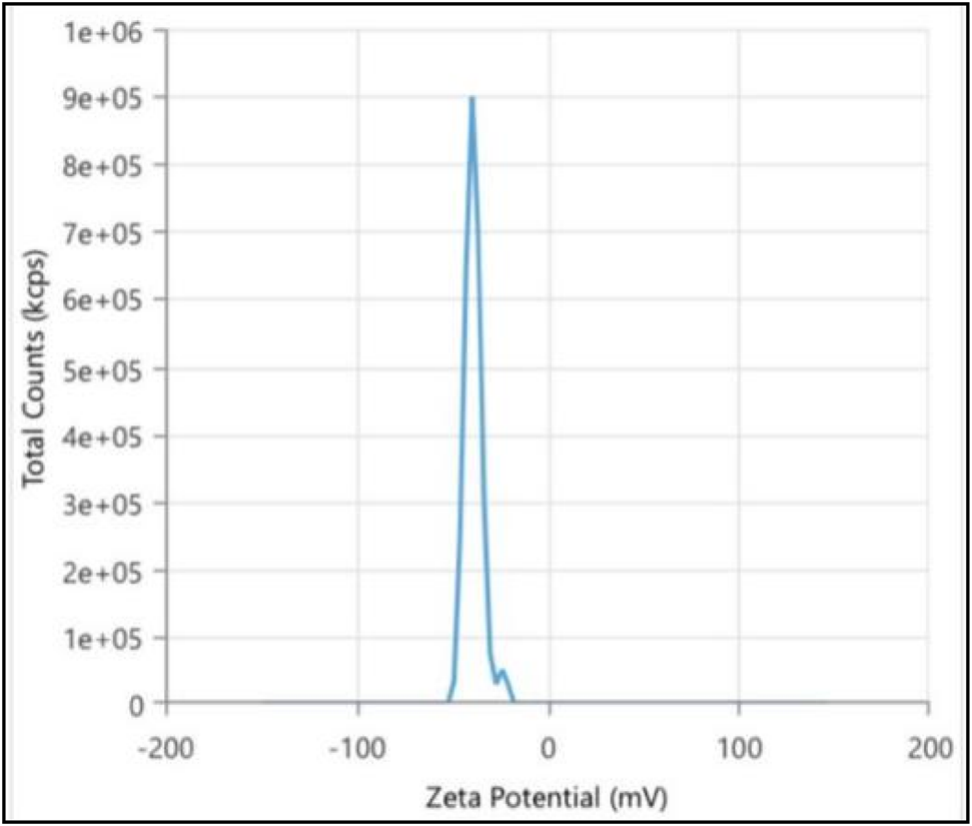
Zeta potential analysis of AgNPs: graph represents the zeta potential of −38.61 mV, indicating surface charge and colloidal stability.

The biogenic AgNPs in this study exhibited a zeta (*ζ*) potential of −38.61 mV Fig. 8, which reflects strong electrostatic stability. Typically, NPs with zeta potentials beyond ±30 mV are considered stable in colloidal dispersions and the *ζ* potential is therefore recognized as a critical factor in evaluating the stability of NPs in solution.^72^ The higher the negative or positive *ζ* potential, the higher the stability, the better the colloidal properties due to electrostatic repulsion and the higher the dispersity.^73,74^ It is important to note that the *ζ* potential is not a fixed property but varies with factors like pH, ionic strength and the dispersion medium, meaning the values here reflect the stability of AgNPs under our experimental conditions. The negative charge observed on the surface of the AgNPs are hence directly linked to the phytochemicals present in the polyherbal extract used.^75^ While some dual-plant systems approach good stability (−34 mV with *Zingiber officinale* & *Allium sativum* and −37 mV with *Zingiber officinale* & *Capsicum annuum*),^76^the zeta potential of the proposed polyherbal AgNPs (−38.61 mV) is more negative than values reported for most single- and dual-plant systems ranging −15 to −37 mV (SI Table 11), indicating excellent electrostatic stabilization and long-term colloidal stability.

#### Fourier transform infrared (FTIR) spectroscopy

**Fig. 9:**
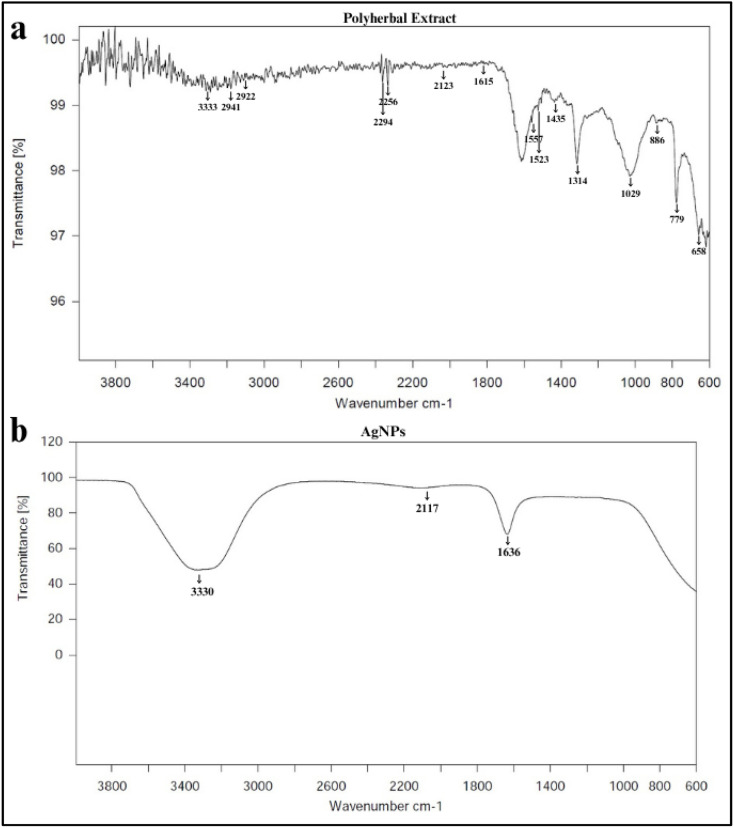
Fourier transform infrared (FTIR) spectra: (a) polyherbal extract spectrum and (b) AgNPs spectrum.

FTIR analysis of the polyherbal extract and synthesized AgNPs revealed distinct spectral changes, confirming the involvement of phytochemicals in AgNPs formation and stabilization. The FTIR spectrum of the polyherbal extract exhibited multiple peaks with the prominent O–H/N–H stretching band at 3333 cm^−1^ shifting to 3329 cm^−1^ in the AgNPs, indicating that hydroxyl and amine groups played a key role in reducing Ag^+^ ions and stabilizing the NPs, as observed in the Fig. 9. Furthermore, a slight shift from 2123 to 2117 cm^−1^ without significant peak intensity changes was observed, reflecting a minor change in the surrounding functional groups or interference from atmospheric CO_2_. The most significant change was observed where the band at 1615 cm^−1^ in the extract shifted to 1635 cm^−1^ in the AgNPs suggesting that carbonyl groups associated with the amide-I band participate in AgNPs stabilization, with possible overlap from the H–O–H bending region.^77^ Previous reports indicate that absorption in the 1700–1600 cm^−1^ region corresponds to biomolecules capping the AgNPs.^78–80^ The attenuation and shift of hydroxyl, carbonyl and amide bands in the AgNPs FTIR spectrum confirm the role of polyphenols, flavonoids and proteins from the polyherbal extract in the reduction and stabilization of AgNPs. This marked reduction in FTIR spectral complexity from the polyherbal extract to the AgNPs characterized by selective retention of –OH/N–H, amide-I bands and suppression of aliphatic, polysaccharide-related signals suggest controlled phytochemical involvement rather than excessive organic overcoating unlike some reported mixed and single plant extract systems (SI Table 11). It is hence suggested that the proposed polyherbal system retains limited key functional groups, indicating selective and cooperative involvement of effective reducing and capping agents.

#### Elemental analysis by energy dispersive X-ray (EDX) spectroscopy

**Fig. 10:**
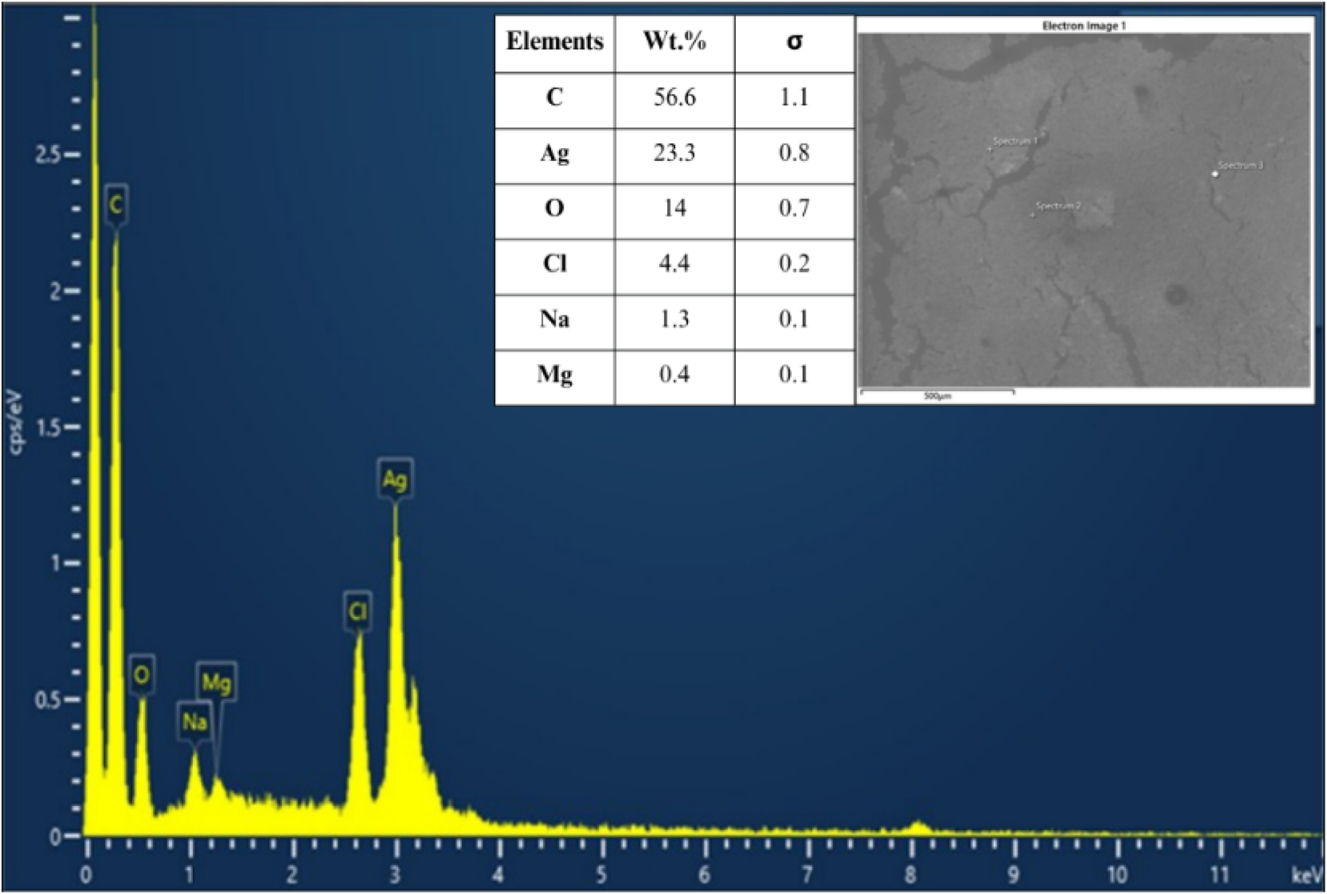
Elemental analysis of AgNPs: SEM-EDX showing elemental composition.

**Fig. 11:**
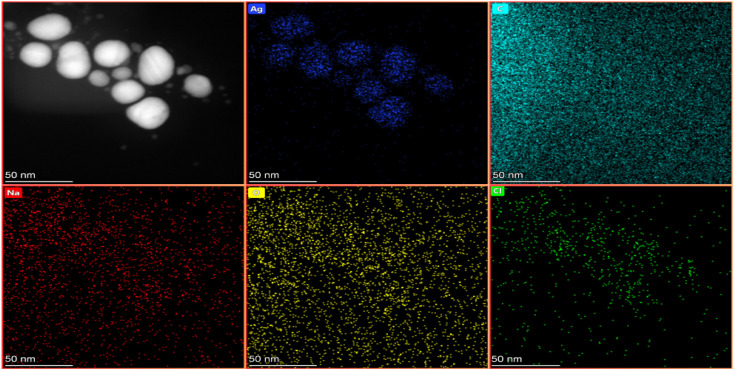
Elemental analysis of AgNPs: TEM-EDS mapping of element distribution.

To determine the elemental composition of the synthesized AgNPs and identify the major elements involved in their formation, SEM-EDX analysis was carried out. The presence of elemental Ag (23.3 wt%) at around 3 keV is associated with the Ag-Lα line,^81^ depicted in Fig. 10. Therefore, EDS analysis validates the successful formation of AgNPs. Additionally, considerable amount of carbon (56.6 wt%) and oxygen (14.0 wt%) was also detected, which can be attributed to phytochemicals in the polyherbal extract. Compounds such as phenolics, flavonoids, polysaccharides, organic acids, amino acids and proteins may serve as natural primary reducing agents, while their functional groups such as hydroxyl (–OH), carboxyl (–COOH) and carbonyl (C=O) contributed to the capping and stabilization of the AgNPs.^79^ Minor elements were also observed, with chlorine (Cl = 4.4 wt%), sodium (Na = 1.3 wt%) and magnesium (Mg = 0.4 wt%) likely originating from the plant metabolites. To confirm the findings, TEM-EDX (Fig. 11) was also performed, which showed the same elemental components with a similar composition as observed in SEM-EDX. This consistency between the two techniques strengthens the reliability of the elemental analysis of the synthesized AgNPs. The detection of these elements highlights the involvement of phytochemicals in the synthesis pathway and further accounts for the stability, biocompatibility and functional properties of the synthesized AgNPs.^82^

#### Size and shape analysis by TEM & DLS

**Fig. 12:**
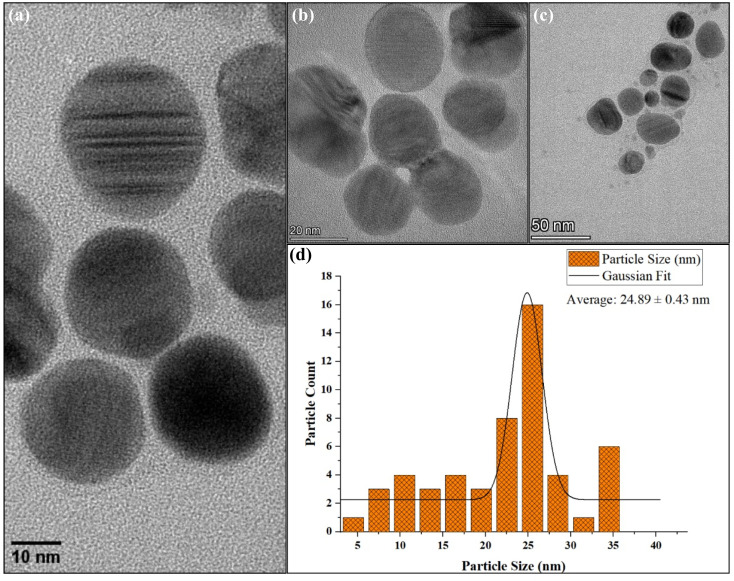
Size and shape analysis of AgNPs: (a–c) TEM showing morphology 10–50 nm and (d) graph representing average particle size of AgNPs ∼25 nm.

**Fig. 13:**
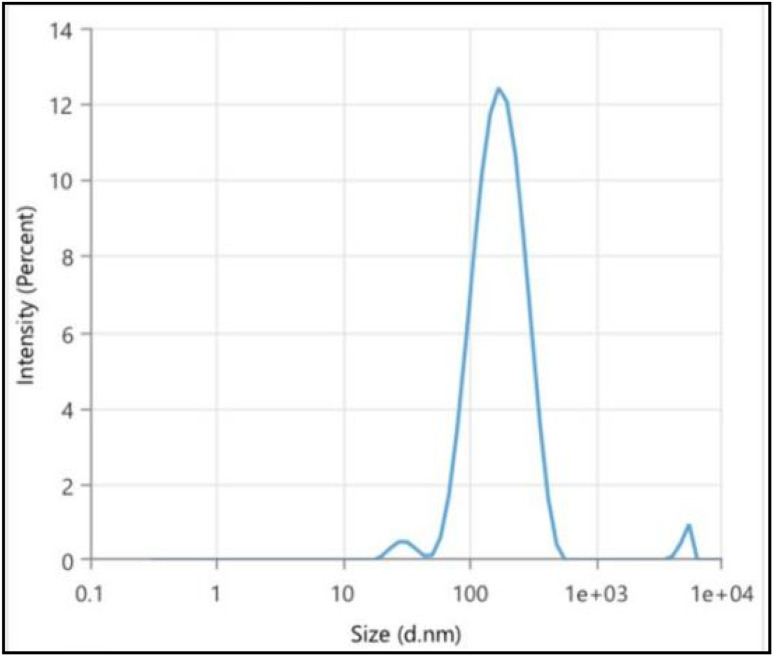
Dynamic light scattering (DLS): hydrodynamic size distribution of AgNPs.

The synthesized AgNPs appeared small, predominantly spherical as observed in Fig. 12a–c and uniformly dispersed, with no significant agglomeration as observed by TEM. This morphology is in good agreement with the nanosphere peak obtained from UV-Vis spectroscopy. They exhibited a narrow size distribution with an average diameter of 25 nm, indicating the successful formation of moderately monodisperse nanoscale Ag with controlled morphology Fig. 12d. Whereas, DLS analysis Fig. 13 revealed an average *H*_D_ of 149.3 nm with a PDI of 0.32, suggesting a moderate size distribution. Here, the larger average size measured by DLS is due to the phytochemicals from the extract coating the AgNPs surface as well as some level of particle clustering may occur in suspension. Since DLS measures NPs in their hydrated state, including the surrounding surface coating and small aggregates therefore, the sizes obtained are generally larger than those observed by TEM, which reveals only the solid metallic core. It is assumed that the presence of the coating makes the NPs more biocompatible. Similar differences between TEM and DLS particle sizes have also been reported in plants like *Ducrosia anethifolia*^83^ and *Rubus discolor*.^84^

#### Crystallinity studies by XRD and SAED

**Fig. 14:**
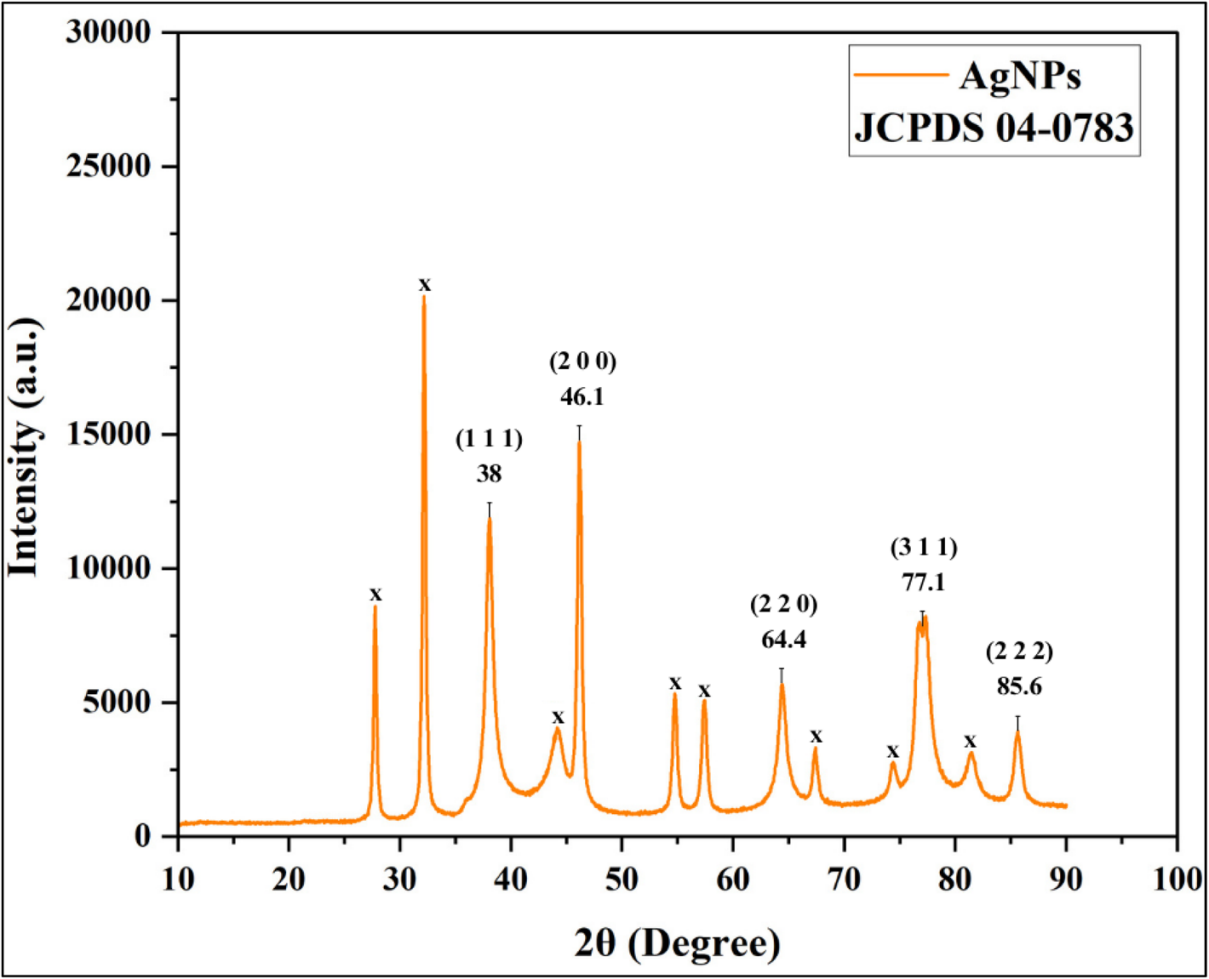
XRD pattern of AgNPs: diffraction peaks corresponding to the (1 1 1), (2 0 0), (2 2 0), (3 1 1) and (2 2 2) planes of face-centered cubic Ag.

**Fig. 15:**
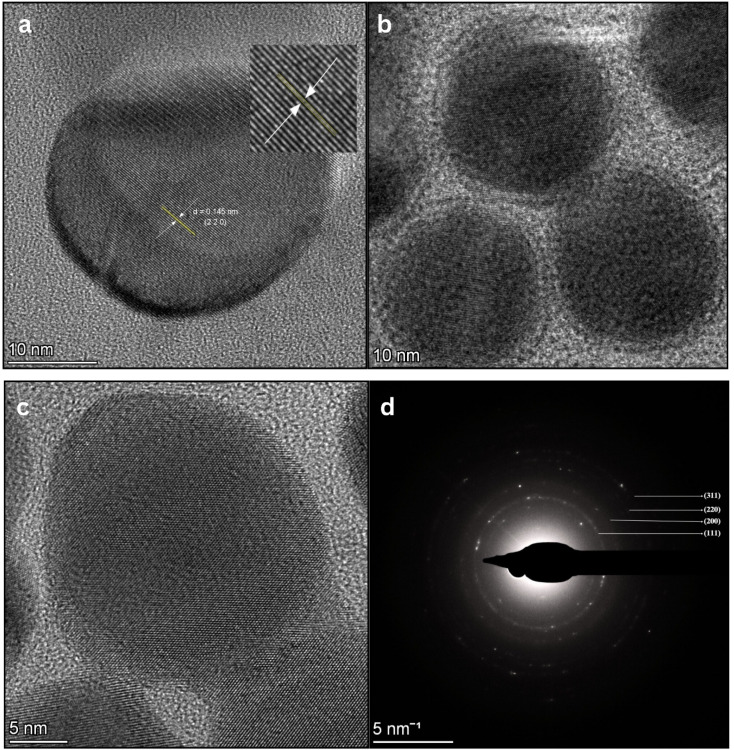
Crystallographic analysis of AgNPs: (a) high-resolution TEM (HR-TEM) image displaying clear lattice fringes with an interplanar spacing (d-value); (b and c) HR-TEM images showing lattice fringes and (d) SAED pattern confirming crystallinity of AgNPs.

The synthesized AgNPs were characterized using XRD, HR-TEM and SAED to assess their structural features. XRD analysis exhibited well-resolved diffraction peaks at 2*θ* values of 38°, 46.1°, 64.4°, 77.1° and 85.6°, which were indexed to the (1 1 1), (2 0 0), (2 2 0), (3 1 1) and (2 2 2) crystallographic planes of face-centered cubic (FCC) Ag, consistent with JCPDS card no. 04-0783. Here, the strongest reflection at 38.1° corresponds to the (1 1 1) plane, indicating preferential growth along this direction, a characteristic commonly reported for metallic Ag nanocrystals (Fig. 14; SI Table 6). Similar observations have been reported for *Rauvolfia serpentina* Benth^85^ and *Garcinia imberti* Bourd.^86^ The appearance of additional peaks in the XRD pattern can be attributed to residual organic compounds originating from the biological extract. Using the Debye–Scherrer equation, the average crystallite size was calculated to be 11.19 nm (Table 2). The equation is expressed as *D* = 0.9 × *λ*/(*β* × cos *θ*), where *D* is the crystallite size, *λ* is the X-ray wavelength (1.541862 Å), *β* is the full width at half maximum (FWHM) of the diffraction peak and *θ* is the Bragg angle. Following this, HR-TEM imaging revealed distinct lattice fringes attributed to the (2 2 0) planes, with an interplanar spacing of 0.145 nm, demonstrating high crystallinity at the nanoscale level (Fig. 15a–c). Furthermore, the SAED concentric rings pattern as illustrated in Fig. 15d, displayed concentric rings with intense spots corresponding to the (1 1 1), (2 0 0), (2 2 0) and (3 1 1) reflections. This confirms the FCC lattice structure of the biogenic AgNPs and indicates their polycrystalline morphology.^87,88^ These findings align well with earlier reports on green-synthesized AgNPs, including those prepared using *Lysiloma acapulcensis*^89^ and *Mitragyna parvifolia*^90^ collectively support the presence of a highly ordered atomic framework in the NPs studies. Therefore, based on the combined TEM, XRD crystallite size and SAED results, it can be inferred that the polyherbal AgNPs are polycrystalline in nature, where multiple crystalline domains constitute a single particle, explaining the smaller crystallite size (11.19 nm) compared to the larger (∼25 nm) TEM core size.

**Table 2:** Crystallite size of AgNPs from XRD analysis

S. no.	Peak position (2*θ*°)	Planes (*hkl*)	FWHM (2*θ*°)	Crystallite size (nm)	Average crystallite size (nm)
1	38.08	−111	0.93	9.04	11.19
2	46.17	−200	0.50	17.26	
3	64.44	−220	0.97	9.70	
4	77.11	−311	1.65	6.18	
5	85.63	−222	0.79	13.78	

#### Stability of colloidal AgNPs

**Fig. 16:**
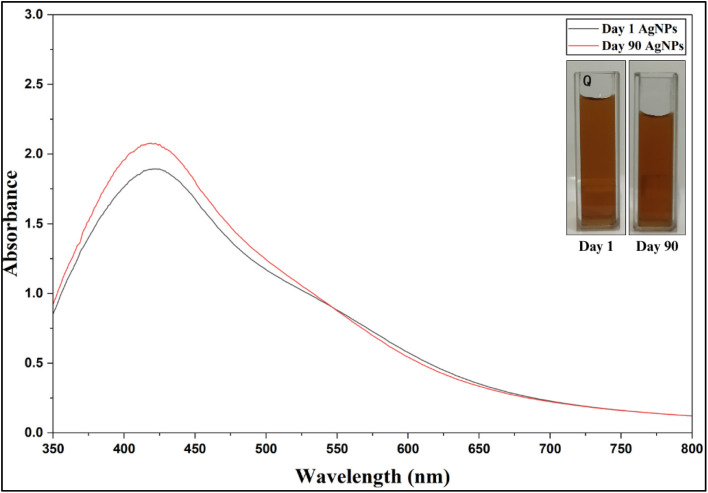
Stability assessment of AgNPs: UV-Vis spectra recorded from day 1 (peak recorded at 422 nm) to day 90 (peak recorded at 422 nm) and the photograph of the colloidal solution retaining its colour.

The UV-visible spectra showed a minor blue shift in the absorption maximum, changing from 422 nm to 419 nm over a period of 90 days, while the absorbance values remained nearly constant with a minor shift of 0.2. No evidence of agglomeration was observed and also there was no visible change in colour; the solution remains bright orange throughout the storage period (Fig. 16; SI Table 7).

### Applications

#### Catalytic activity

The catalytic performance of the synthesized AgNPs was evaluated using the reduction of MB by NaBH_4_ as a model reaction. MB is a widely used phenothiazine dye in biological staining, redox indicators, medicinal applications and industrial colouring; however, its persistence and toxicity upon release into aquatic environments raise significant environmental concerns. In this reaction system, MB is reduced to its leuco form, leuco-methylene blue (leu-MB), which is colourless and considerably less toxic owing to its reduced reactivity and lack of environmental persistence.^91^ The progress of the catalytic reaction was monitored spectrophotometrically. In aqueous solution, MB displays a characteristic absorption maximum at 663 nm and the extent of its degradation can be readily followed by the gradual decrease in the intensity of this absorption band.

**Fig. 17:**
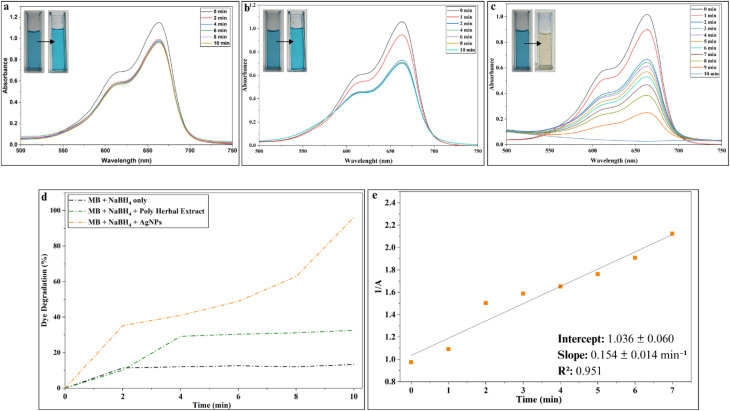
Catalytic potential of AgNPs in methylene blue (MB) degradation: (a) UV-Vis spectra of MB with NaBH_4_ only (1–10 min); (b) UV-Vis spectra of MB with NaBH_4_ and polyherbal extract (1–10 min); (c) UV-Vis spectra of MB with AgNPs, showing rapid degradation; (d) degradation percentage comparison for all three systems; (e) pseudo-second-order kinetic fit for MB degradation with AgNPs.

13% degradation was observed in the presence of NaBH_4_ alone (Fig. 17a; SI Table 8), while 32% degradation occurred when the polyherbal extract was used with NaBH_4_ in the absence of AgNPs (Fig. 17b; SI Table 8). In contrast, the addition of polyherbal AgNPs resulted in rapid and efficient MB degradation, reaching approximately 96% within ten minutes (Fig. 17c; SI Table 8). This partial degradation of MB by polyherbal extract is likely due to the bioactive phytochemicals present in the polyherbal extract, which can induce limited redox activities. However, the introduction of AgNPs substantially enhanced the reaction by facilitating faster electron transfer, resulting in near-complete MB reduction within a much shorter time (Fig. 17d; SI Table 8).

Kinetic analysis based on the linear relationship between 1/*A* and time indicated pseudo-second-order behavior, yielding an apparent rate constant of (1.09 ± 0.10) × 10^4^ L mol^−1^ min^−1^ (Fig. 17e; SI Table 8). The enhanced catalytic activity can be attributed to the role of AgNPs as electron mediators, facilitating rapid electrons transfer from NaBH_4_ to MB. The electron-rich surface of AgNPs lowers the activation barrier and accelerates the reduction process,^92^ while it is also possible that the phytochemicals stabilizing the AgNPs may further promote dye adsorption through hydrophobic interactions and van der Waals forces;^93^ however, this has not been directly confirmed in our system.

Accordingly, the catalytic performance of the present multi-plant-mediated AgNPs was evaluated in comparison with representative single-plant systems reported in the literature. As summarized in SI Table 11, most single-plant-mediated AgNPs show high MB degradation efficiencies (approximately 82–96.7%); however, they typically require longer reaction times (6–120 min) to achieve these levels. However, in our study the AgNPs synthesized using the combined extracts achieved 96% degradation of MB within 10 min, indicating a substantially faster catalytic process. Although the AgNPs in the present study possess a particle size (∼25 nm) comparable to many previously reported systems, they exhibit improved reaction kinetics, with a rate constant of (1.09 ± 0.10) × 10^4^ L mol^−1^ min^−1^. This enhanced performance is likely due to the contribution of diverse phytochemicals from multiple plant sources, which can modify the AgNPs surface and promote more efficient electron transfer during MB reduction. While, the direct comparison with other multi-plant-mediated AgNPs systems is limited by the lack of reported studies. Overall, these findings suggest that the multi-extract synthesis approach offers catalytic performance that is comparable to and in several cases better than, single-plant-mediated AgNPs for MB degradation.

#### Preliminary colorimetric screening of heavy metal ions

**Fig. 18:**
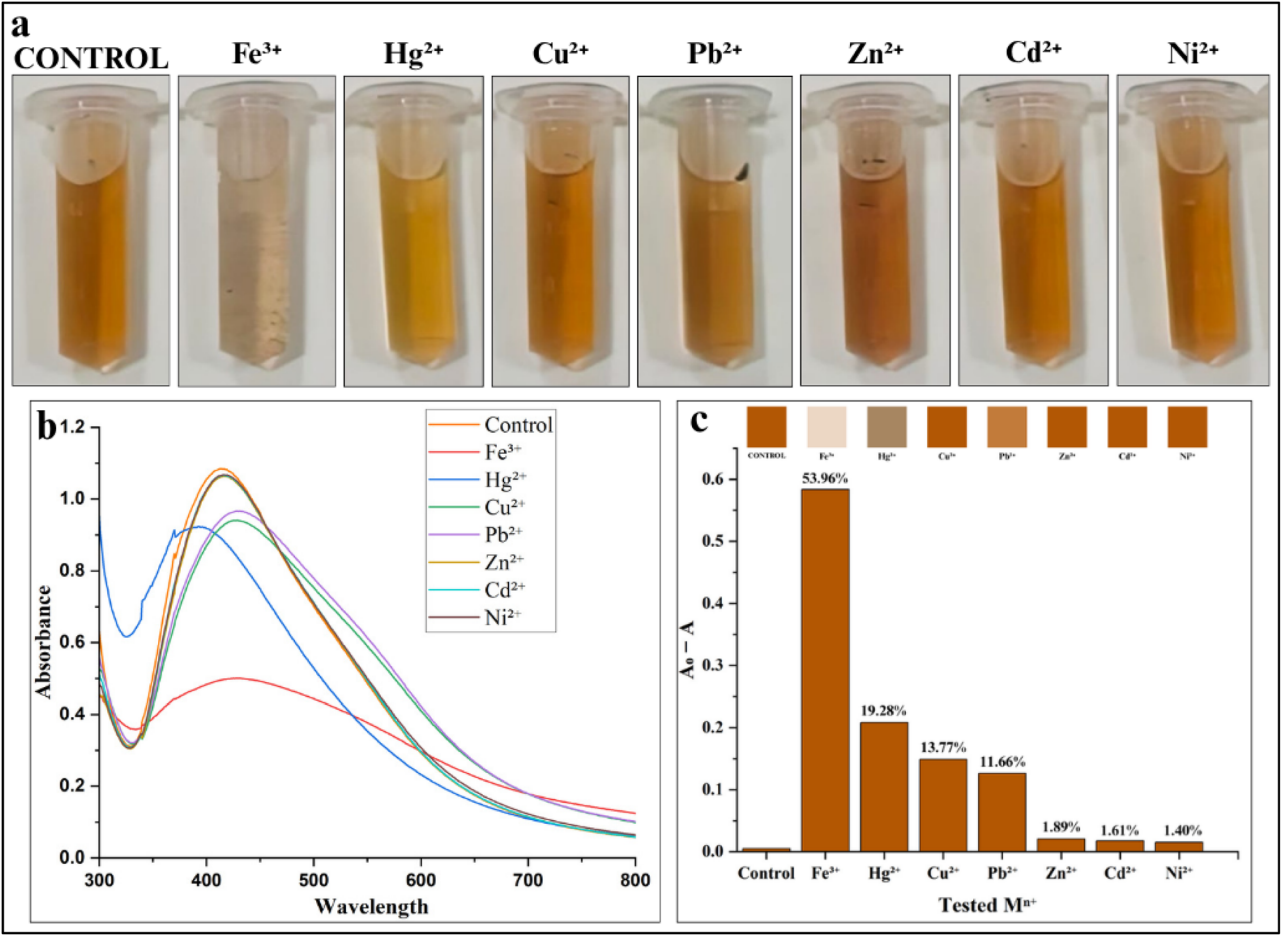
Sensitivity of AgNPs to different heavy metal ions: (a) visual color changes of AgNPs with Fe^3+^; (b) UV-Vis spectra showing the quantitative response against the test heavy metal and (c) distribution of SPR quenching percentage for the corresponding metal ions.

The biosynthesized AgNPs exhibited strong selectivity toward Fe^3+^ ions, showing the highest SPR quenching at 53.96%, accompanied by a colour change from bright-orange to colourless within 30 min (Fig. 18a and b; SI Table 9). Whereas, other tested metals, including Hg^2+^, Zn^2+^, Pb^2+^, Ni^2+^ and Cu^2+^, showed minimal or no change, with SPR quenching ranging from 19.28% to 1.40% respectively (Fig. 18c), highlighting the high specificity of the AgNPs toward Fe^3+^ owing to distinct chemical interactions at the NPs surface, as further confirmed by the colour change. In this system, Fe^3+^ ions were seen to strongly coordinate with the surface functional groups of the AgNPs, promoting aggregation and a pronounced spectral response.^94,95^ Meanwhile, a slight colour change was also observed with Hg^2+^, likely due to its ability to undergo redox interactions with Ag^0^.^56,96^ However, the SPR quenching was only 19.28%, indicating a weaker and less selective interaction than that of Fe^3+^. In contrast, other tested metal ions such as Cu^2+^, Pb^2+^, Zn^2+^, Cd^2+^ and Ni^2+^ did not have sufficient redox potential or surface affinity towards the present study AgNPs to induce comparable aggregation or changes in SPR. Therefore, only Fe^3+^ was selected for the next step of screening. This selective detection of Fe^3+^ is consistent with previous reports, where biogenic AgNPs synthesized using *Croton bonplandianum*^97^ and *Bauhinia variegata*^98^ were reported to selectively detect Fe^3+^ ions.

#### Quantitative sensing of Fe^3+^ ions

**Fig. 19:**
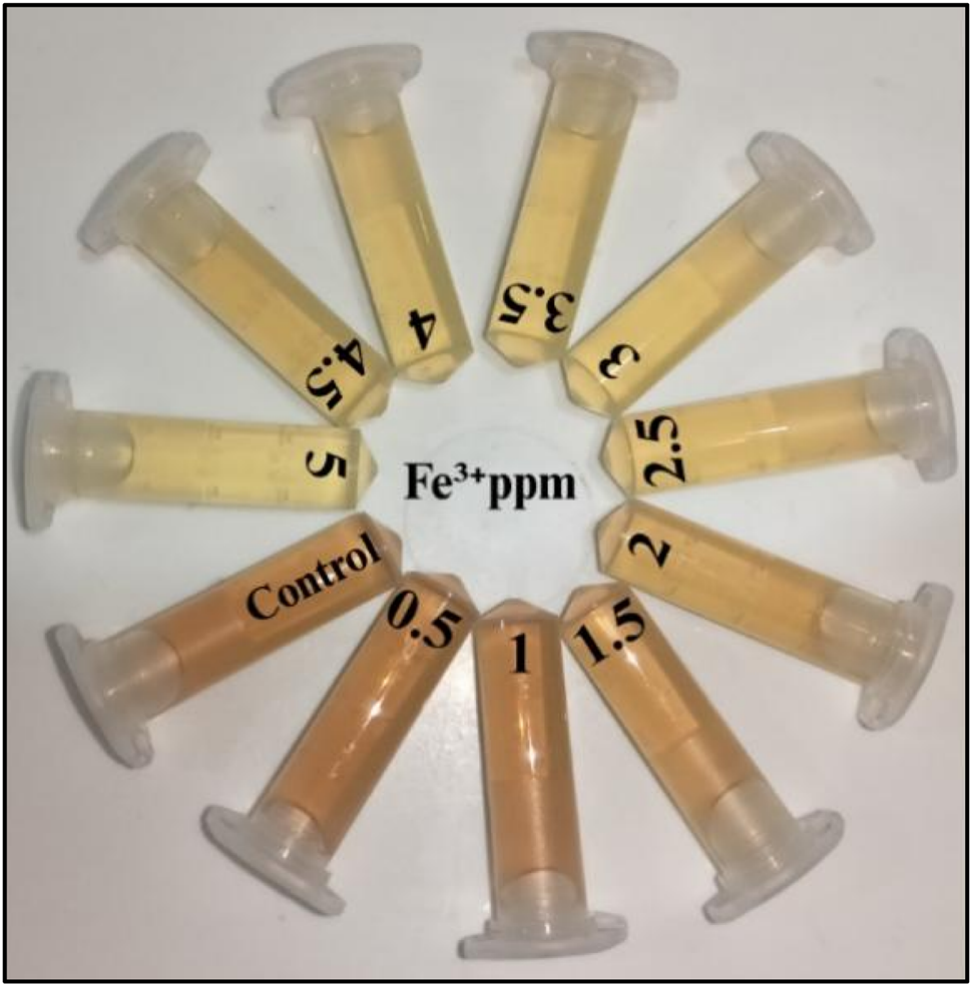
Fe^3+^–AgNPs interactions: visual color change of AgNPs solutions with increasing Fe^3+^ concentration (0.5–5 ppm).

**Fig. 20:**
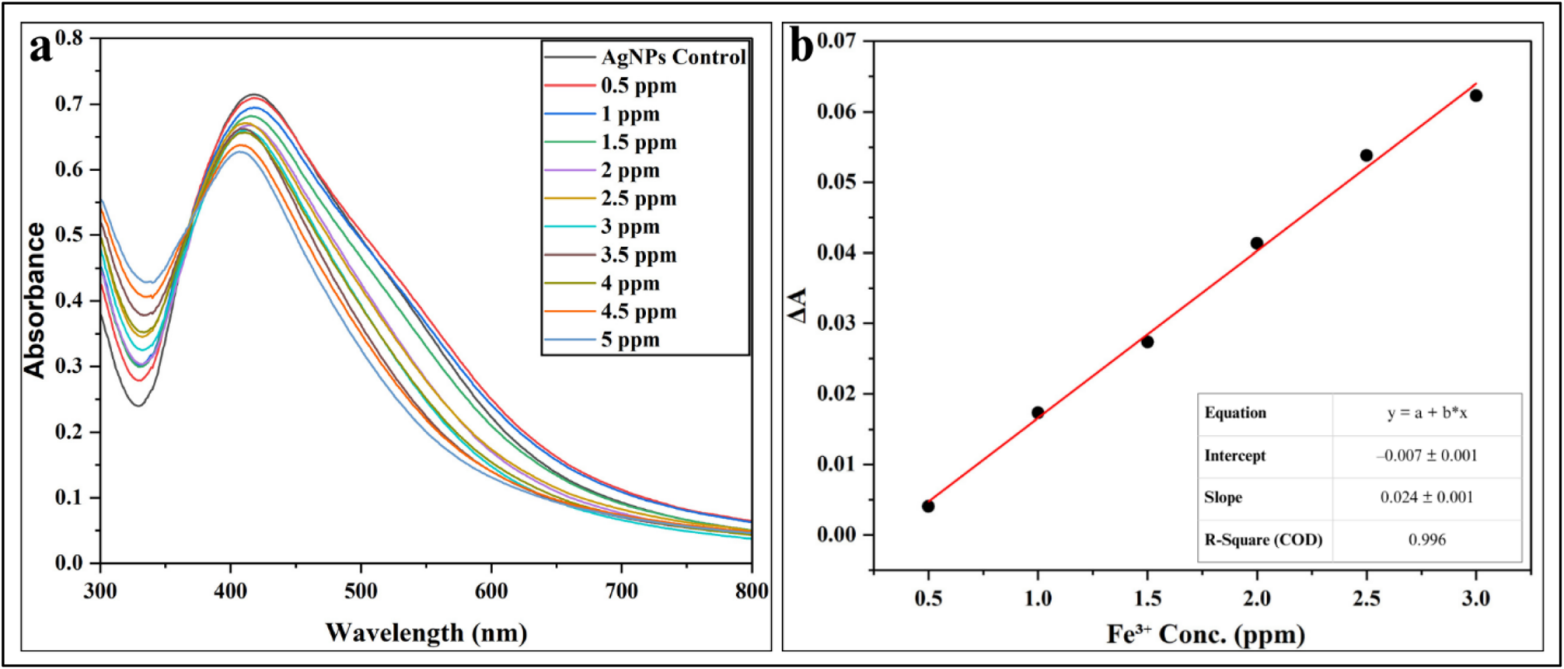
Fe^3+^–AgNPs UV-Vis analysis: (a) decreasing absorbance with blue shift (0.5–5 ppm) and (b) calibration curve of Fe^3+^ concentration *vs.* absorbance change.

For the Quantitative detection of Fe^3+^ ions, colorimetric and absorption spectroscopy were used to study the SPR response of freshly synthesized AgNPs to Fe^3+^ over a range of 0.5–5 ppm. To ensure reliability under ambient conditions, the AgNPs' concentration was kept constant and the absorbance range was maintained between 0.6–0.8. Upon the introduction of Fe^3+^, the AgNPs solution displayed a clear and progressive colour change, shifting from a bright-orange at lower ion concentrations to an almost colourless solution at higher concentrations (Fig. 19). Further, UV-visible absorption spectra (Fig. 20a; SI Table 10) supported these findings, with calibration plots revealing a strong linear relationship in the range of 0.5–3 ppm and a determination coefficient (*R*^2^ = 0.996) (Fig. 20b). A gradual hypochromic shift in the absorption maximum was observed as the Fe^3+^ concentration increased, suggesting alterations in the electronic environment of the AgNPs. The calculated LOD in this study was 0.443 ppm (7.93 µM), underlining the potential of the system for detecting Fe^3+^ at trace levels.

**Fig. 21:**
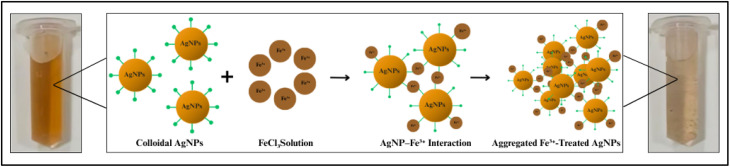
Mechanism of Fe^3+^–AgNPs interaction: AgNPs are stabilized by a green phytochemical capping corona. When Fe^3+^ ions (brown spheres) were added, they interacted with the capping layer and NPs surface, causing aggregation and loss of colloidal stability, resulting in the fading of the characteristic colour (bright-orange to colourless).

The sensing mechanism taking place in this senor involves a redox interaction between AgNPs and Fe^3+^ ions. Here, Fe^3+^ is acting as an oxidizing agent, withdrawing electrons from Ag^0^ from the NPs surface, which are oxidized to Ag^+^, while Fe^3+^ is reduced to Fe^2+^. This electron transfer may disrupt the electronic state of the AgNPs and partially destabilizes the plant extract coating, causing aggregation.^99^ Alternatively, this redox process can also lead to partial etching/dissolution of the AgNPs rather than aggregation. The gradual loss of metallic Ag from the NPs surface decreases particle size and can result a blue shift as observed in this system, accompanied by a visible colour change from bright-orange to colourless along with loss of AgNPs structure with the increase in the concentration of Fe^3+^ (Fig. 21).^100^

In this context, SI Table 3 summarizes previously reported biogenic single plant mediated AgNPs-based colorimetric sensors for Fe^3+^ detection, highlighting the wide variation in sensing performance which can be attributed to the difference in phytochemical composition of selected plants and NPs surface chemistry. Whereas, the present multi-plant-mediated AgNPs system showed a LOD of 0.443 ppm and a strong linear response (*R*^2^ = 0.996) across the concentration range of 0.5–3 ppm. This operating range closely matches the World Health Organization (WHO)^101^ and EPA recommended iron levels (0.3 mg L^−1^), which can lead to a noticeable deterioration in water quality. Overall, these results demonstrate that the multi-plant formulation offers a robust and application-oriented sensing platform, though the direct comparison with other multi-plant-mediated AgNPs systems is limited due to the lack of reported multi-extract Fe^3+^ sensors.

## Conclusion

The present study successfully demonstrates the potential of polyherbal (*Rubia cordifolia*, *Terminalia arjuna* and *Bombax ceiba*) aqueous extract for development of sustainable AgNPs. Spectroscopic and microscopic analyses confirmed the formation of predominantly spherical, polycrystalline AgNPs with an average core size of approximately 25 nm. While, zeta potential measurements (−38.61 mV) and storage studies indicated good colloidal stability under the experimental conditions employed.

The functional performance of the synthesized AgNPs was evaluated using two model systems: (i) catalytic reduction of MB in the presence of NaBH_4_ and (ii) colorimetric detection of heavy metal ions. In the presence of NaBH_4_, the AgNPs catalyzed the reduction of MB, achieving ∼96% degradation within 10 min and following pseudo-second-order kinetics with an apparent rate constant of (1.09 ± 0.10) × 10^4^ L mol^−1^ min^−1^. While the observed degradation efficiency and reaction rate are comparable to and in some cases faster than, several reported single-plant-mediated AgNPs systems. The AgNPs also exhibited a selective colorimetric response toward Fe^3+^ ions among the tested metal ions, as evidenced by pronounced SPR quenching and a visible colour change. Quantitative analysis showed a linear response in the range of 0.5–3 ppm (*R*^2^ = 0.996) with a calculated detection limit of 0.443 ppm. Altogether, this study highlights the feasibility of multi-plant green synthesis as an environmentally compatible route for producing functional AgNPs, which possess reproducible physicochemical characteristics and exhibit dual functionality in model catalytic and sensing applications.

## Author contributions

Ayushma Vyavahare: formal analysis, investigation, resources, visualization, writing original draft. Priyanshi Dhiraj Shah: formal analysis, investigation, methodology, resources, visualization, validation, writing original draft. Vivek Mishra: formal analysis, investigation, methodology. Abuzar Shakil Patel: formal analysis, investigation, methodology. Vasundhara Raina: conceptualization, methodology, supervision, writing original draft, review & editing. Sarmita Sanjay Jana: conceptualization, methodology, supervision, writing original draft, review & editing.

## Conflicts of interest

The authors declare that there are no conflicts of interest regarding the publication of this paper. The research was conducted in the absence of any commercial or financial relationships.

## Supplementary Material

RA-016-D5RA08581K-s001

## Data Availability

All data generated or analysed during this study including the experimental data, spectral analyses and figure datasets supporting the findings are provided as addendum materials in this article and its supplementary information (SI) files. The authors have cited additional references (Ref. 102–106) within the SI. Supplementary information is available. See DOI: https://doi.org/10.1039/d5ra08581k.
